# Evaluation of offset of conjunctival hyperemia induced by a Rho-kinase inhibitor; 0.4% Ripasudil ophthalmic solution clinical trial

**DOI:** 10.1038/s41598-019-40255-9

**Published:** 2019-03-06

**Authors:** Emi Sakamoto, Waka Ishida, Tamaki Sumi, Tatsuma Kishimoto, Kentaro Tada, Ken Fukuda, Tsuyoshi Yoneda, Hajime Kuroiwa, Etsuko Terao, Yasuko Fujisawa, Shunsuke Nakakura, Koji Jian, Hideaki Okumichi, Yoshiaki Kiuchi, Atsuki Fukushima

**Affiliations:** 1 Department of Ophthalmology and Visual Science, Kochi Medical School, Kochi University, Nankoku City, Kochi Japan; 2 Department of Ophthalmology, Kawasaki University of Medical Welfare, Kurashiki City, Okayama Japan; 3 Integrated Center for Advanced Medical Technologies, Kochi Medical School, Kochi University, Nankoku City, Kochi Japan; 4 Department of Ophthalmology, Saneikai Tsukazaki Hospital, Himeji City, Hyogo Japan; 5 Department of Ophthalmology and Visual Sciences, Graduate School of Biomedical Sciences, Hiroshima University, Hiroshima City, Hiroshima Japan

## Abstract

Glaucoma leads to irreversible blindness. Numerous anti-glaucoma eye drops have been developed. Unfortunately, many patients with glaucoma still suffer from progressive visual disorders. Recently, ripasudil hydrochloride hydrate, a selective Rho-associated protein kinase inhibitor, was launched for the treatment of glaucoma. However, adverse events, such as conjunctival hyperemia, are often noted in clinical trials using healthy subjects. Therefore, we investigated the onset, offset, and kinetic changes of conjunctival hyperemia induced by ripasudil ophthalmic solution in patients with open-angle glaucoma or ocular hypertension who had already been treated with anti-glaucoma eye drops other than ripasudil. Conjunctival hyperemia was evaluated by both clinical grading by 3 ophthalmic physicians and pixel coverage of conjunctival blood vessels determined by conjunctival hyperemia-analyzing software. Conjunctival hyperemia appeared within 10 min post-instillation in most of the participants. Clinical grade and pixel coverage increased significantly 10 min post-instillation and then decreased. In most of the participants, hyperemia resolved within 2 h. Median conjunctival hyperemia offset was 90 min. A tendency of monotonic increase was observed between clinical grade and pixel coverage. Taken altogether, hyperemia induced by ripasudil was transient in glaucoma patients who had already been treated with anti-glaucoma eye drops other than ripasudil.

## Introduction

Glaucoma can cause irreversible blindness, so numerous anti-glaucoma eye drops have been developed. Unfortunately, many patients with glaucoma still suffer from progressive visual defects and vision disorders. There are various types of glaucoma, and it is known to be a multifactorial disease. The most important risk factor for progression is intraocular pressure (IOP), followed by aging, family history, myopia, and low cerebrospinal fluid pressure. At the present, there are no effective treatments other than lowering IOP by instilling eye drops, surgery, or laser procedures.

Rho-associated protein kinase (ROCK) inhibitors lower IOP^1–3^ and are also associated with adverse events; most frequently, conjunctival hyperemia^1–3^. A phase 1 clinical trial of a selective ROCK inhibitor (K-115) found kinetic changes in conjunctival hyperemia at 0.5, 2, 4, 8, and 9 h post-instillation in 50 healthy volunteers^4^. Conjunctival hyperemia was observed at 30 min post-instillation at different K-115 concentrations (0.05%, 0.1%, 0.2%, 0.4% and 0.8%). IOP decreased 1–2 h after a single instillation of K-115. Slight to mild conjunctival hyperemia was found in more than half of the participants treated with K-115, after each instillation, and spontaneously resolved within 4 h. Further, conjunctival hyperemia was observed at various time-points and concentrations, and resolved spontaneously within 4 h, except for 0.8% ripasudil. However, changes in conjunctival hyperemia, observed up to 30 min post-instillation, have not been evaluated.

In December 2014, ripasudil hydrochloride hydrate, a selective ROCK inhibitor (GLANATEC^R^, ophthalmic solution 0.4%: K-115, KOWA Company. Ltd., Nagoya, Japan) was launched as an anti-glaucoma treatment in Japan. A recent clinical trial with 51 healthy participants investigated changes in conjunctival hyperemia and reduction of IOP within 2 h (at 5, 15, 30, 60, 120 min) post-instillation of 0.4% ripasudil^5^. Here, conjunctival hyperemia peaked 5–15 min post-instillation and resolved within 2 h. IOP values decreased between 30 min and 2 h post-instillation, regarding healthy participants. However, ripasudil-induced changes in conjunctival hyperemia over time have not been investigated in patients with glaucoma, who were previously treated with anti-glaucoma eye drops, other than ripasudil. In the present study, we evaluated the offset of conjunctival hyperemia, induced by ripasudil, in patients with open-angle glaucoma or ocular hypertension (OHT).

## Results

### Study Participants

A total of 50 participants were enrolled in this trial, between September 16, 2015 and June 30, 2017. Demographic variables including sex, type of glaucoma, and prior medications are shown in Table 1.

**Table 1:** Participants’ characteristics.

	Primary endpoint	Secondary endpoint
**Age**	67.71 ± 13.87	68.06 ± 13.33
**Sex**		
Male	16	22
Female	26	28
**Types of glaucoma**		
Open angle glaucoma	40	48
Ocular hypertension (OHT)	2	2
**Component of prior instillation**		
Mono (β)	4	5
Mono (PG)	26	32
2 components (PG + β)	6	7
2 components (PG + CAI)	1	1
3 components (PG + β + CAI)	5	5

PG, prostaglandin analogs; β, β-blocker; CAI, carbonic anhydrase inhibitors.

### Primary endpoint

**Figure 1:**
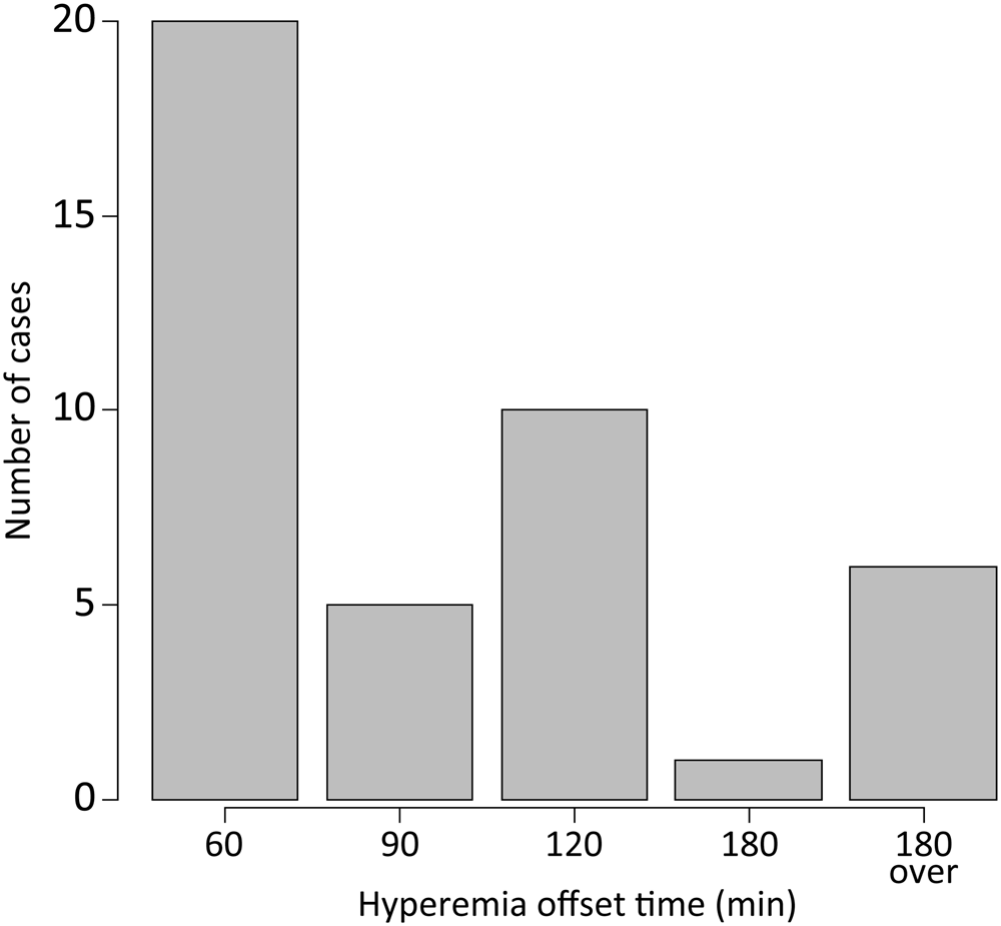
Primary endpoint of conjunctival hyperemia offset time based on clinical grade. The horizontal axis represents the offset time of conjunctival hyperemia induced post-instillation of ripasudil. The vertical axis represents the number of cases within each group. The median time conjunctival hyperemia offset, as per clinical grading, was 90 min and the 95% confidence interval was 60 to 120 min post-instillation of ripasudil.

Clinical grades were used to determine the presence of, and changes in, conjunctival hyperemia. For the primary endpoint, we excluded 8 participants because were unable to induce conjunctival hyperemia by ripasudil. Therefore, data from 42 cases (16 males and 26 females) were analyzed. The mean age was 67.7 years (range: 24–86 years). The median offset time of conjunctival hyperemia, according to clinical grade, was 90 min, with a 95% confidence interval of 60 to 120 min post-instillation of ripasudil. Most frequently, conjunctival hyperemia disappeared within 60 min post-instillation (Fig. 1).

In the group with the offset time within 60 min, the proportion of the participants that had been treated with prostaglandin analogs (PG) alone (PG Mono) was significantly higher than that with β-blocker (Table 2). On the contrary, in the group with the offset time of 180 min or over, the proportion of the participants that had been treated with β-blocker was significantly higher than that with PG Mono (Fisher’s exact test, *P* = 0.02) (Table 2).

**Table 2:** Comparison of prior treatments regarding the offset time.

Prior treatments	Offset time within 60 min	Offset time of 180 min or over
PG Mono	17 case	2 case
β-blocker (Mono, β + PG, β + PG + CAI)	3 case	4 case

PG, prostaglandin analogs; β, β-blocker; CAI, carbonic anhydrase inhibitors.

Fisher’s exact test: *P* = 0.02.

### Secondary endpoints

**Figure 2:**
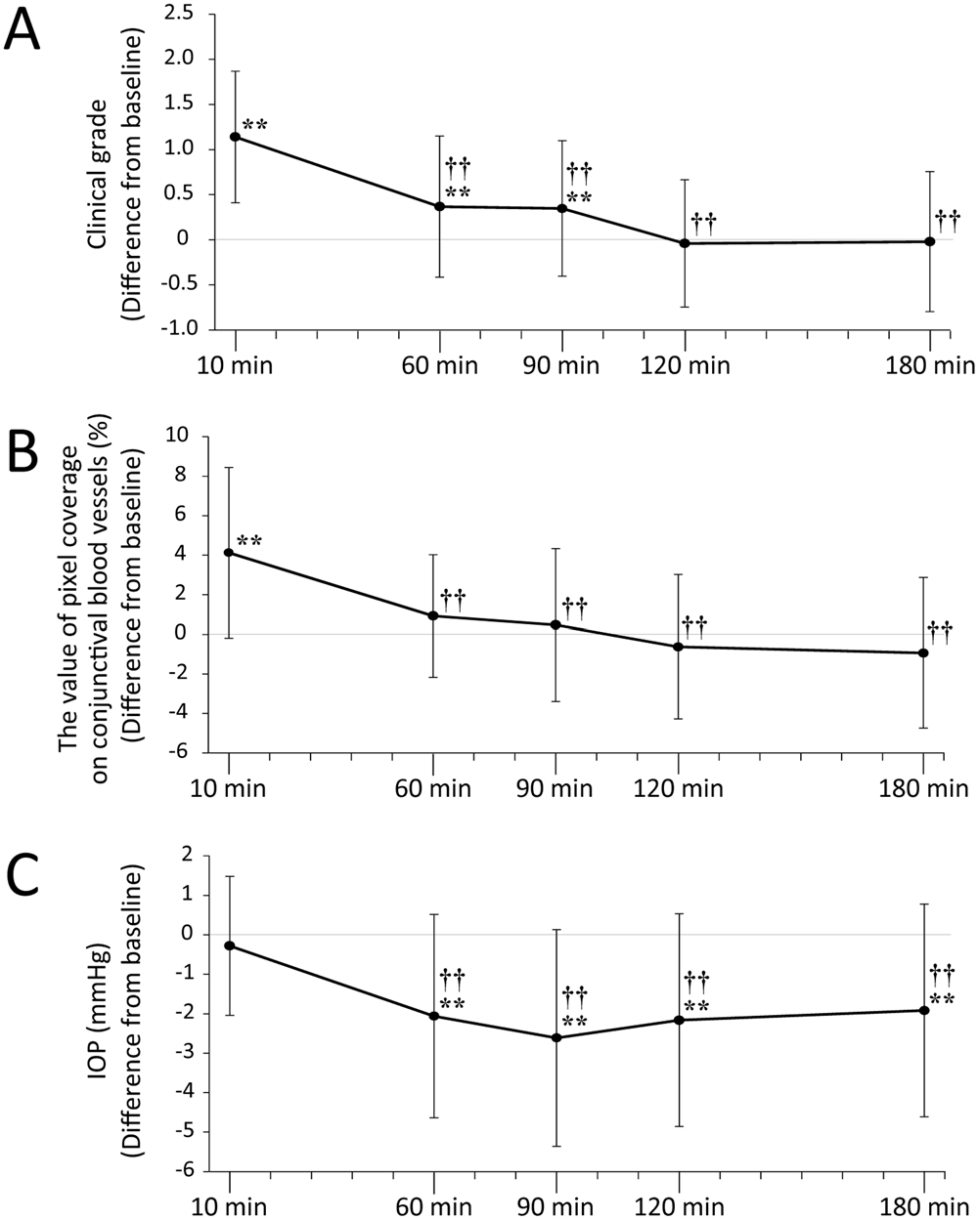
The kinetic changes of clinical grade score, the value of pixel coverage on conjunctival blood vessels, and IOP measurements. The vertical axis of the upper graph shows the mean difference from baseline ± SD regarding clinical grade at each time point. The clinical grade peaked 10 min post-instillation and was at its minimum 120 min post-instillation (**A**). The vertical axis of the middle graph shows the mean difference from baseline ± SD regarding value of pixel coverage on conjunctival blood vessels (%). Pixel coverage values peaked 10 min post-instillation and was at its minimum 180 min post-instillation (**B**). The vertical axis of the lower graph shows the mean difference from baseline ± SD regarding IOP value at each time point. IOP decreased significantly at all time points after 60 min, compared to IOP values at baseline. The lowest IOP value was 90 min post-instillation (**C**). **P < 0.01 (Paired t-test) versus the corresponding value for before instillation as baseline, ^††^*P* < 0.01 (Paired t-test) versus the corresponding value for 10 min post-instillation.

Differences compared with baseline data regarding clinical grade, the value of pixel coverage on conjunctival blood vessels, and IOP values within 180 min post-instillation are shown in Fig. 2. Clinical grade score significantly increased at 10 min, 60 min, and 90 min post-instillation compared with baseline (**P < 0.01). Then, compared to 10 min post-instillation, clinical grade score significantly decreased after 60 min, 90 min, 120 min, and 180 min (^**††**^*P* < 0.01). The clinical grade score was minimal at 120 min post-instillation (Fig. 2A).

The value of pixel coverage on conjunctival blood vessels significantly increased at 10 min post-instillation compared with baseline (**P < 0.01). Then, compared to 10 min post-instillation, the value of pixel coverage significantly decreased after 60 min, 90 min, 120 min, and 180 min (^**††**^*P* < 0.01). The value of pixel coverage was minimal at 180 min post-instillation (Fig. 2B).

Compared to baseline, IOP decreased after 60 min, 90 min, 120 min and 180 min, and significant differences were noted except for 10 min (**P < 0.01). Then, compared to 10 min post-instillation, IOP decreased after 60 min, 90 min, 120 min, and 180 min (^**††**^*P* < 0.01) (Fig. 2C).

**Figure 3:**
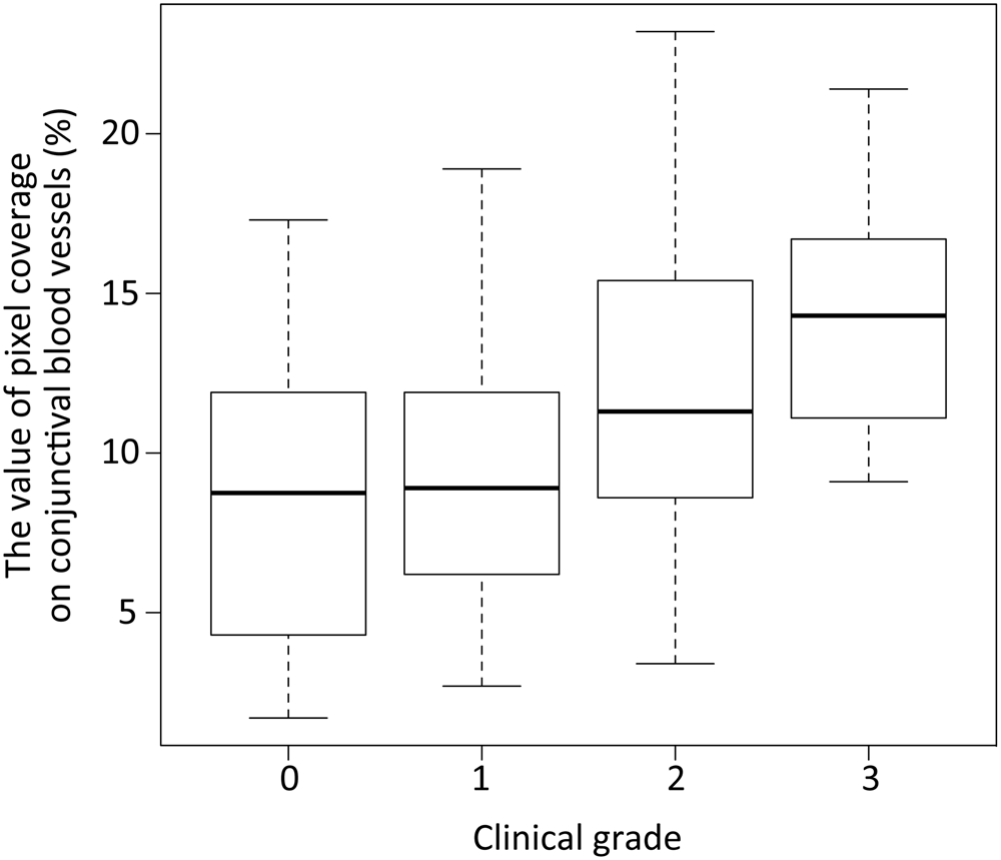
The correlation between clinical grade and pixel coverage value on the conjunctival blood vessels. This box-whisker plot represents the correlation between clinical grades and pixel coverage values on conjunctival blood vessels (%). For both variables, there were monotonic increases, and a one-tailed test confirmed a significant difference (*P* < 0.01, Jonckheere-Terpstra Trend Test, one-tailed test).

A tendency of monotonic increase was observed between clinical grade and pixel coverage of conjunctival blood vessels (*P* < 0.01, Jonckheere-Terpstra Trend Test, one-tailed test) (Fig. 3).

**Figure 4:**
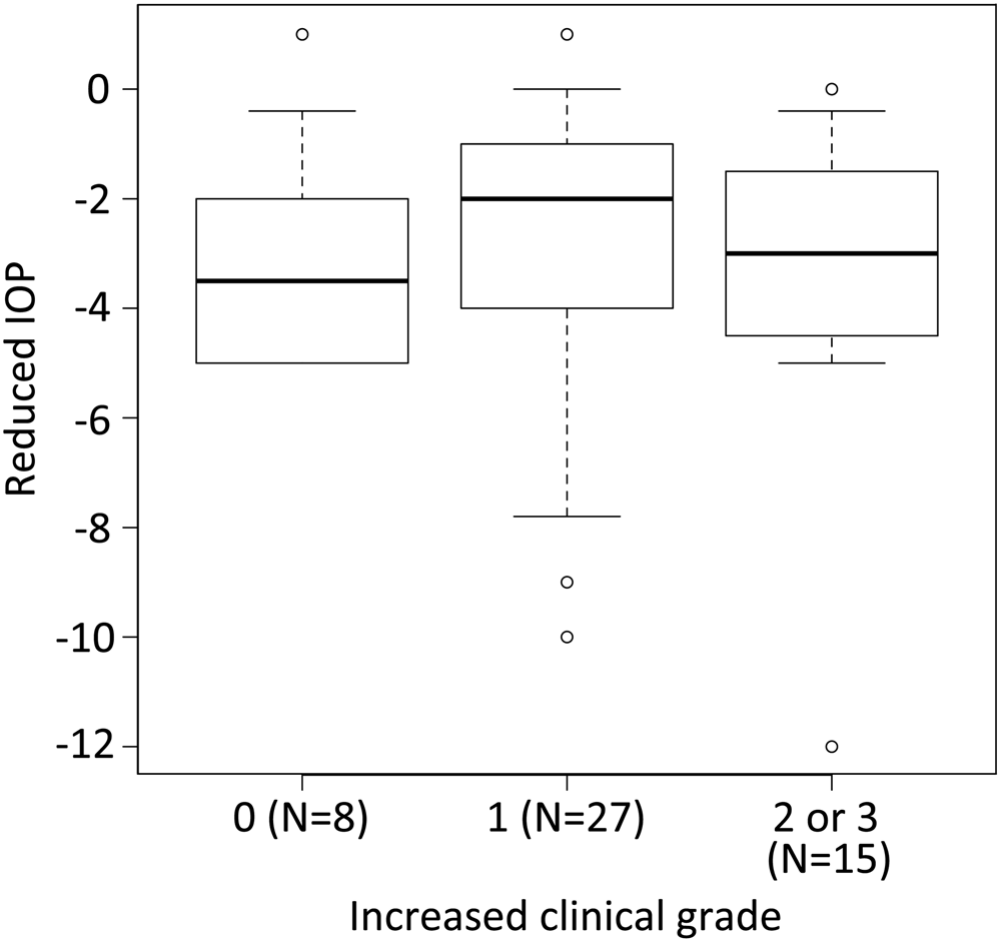
The correlation between increased clinical grade and reduced IOP. This box-whisker plot depicts the correlation between increased clinical grade and reduced IOP (mmHg). The horizontal axis indicates clinical grade (the maximum clinical grade score observed post-instillation minus baseline clinical grade score), and the vertical axis represents the reduction of IOP (the minimum of IOP post-instillation) − (the IOP of baseline). For correlations between clinical grade and IOP, there was a non-significant trend *P* = 0.896 (Jonckheere-Terpstra Trend Test, two-tailed test). IOP values varied widely, and outliers at the top and bottom of the box-and-whisker plot are indicated by circles.

IOP was lowest at 90 min post-instillation. No correlation was observed between increases in clinical grade and reductions in IOP (*P* = 0.896, Jonckheere-Terpstra Trend Test, two-tailed test) (Fig. 4).

## Discussion

Several reports have examined correlations between anti-glaucoma ophthalmic solutions and conjunctival hyperemia. Honrubia *et al*. reported that the incidences of hyperemia related to use of prostaglandin analog ophthalmic solutions were 3.3% to 71.4% (bimatoprost > travoprost > latanoprost)^6^. Using automated hyperemia analysis software^7^, Yanagi *et al*. compared the severity of hyperemia resulting from use of various types of anti-glaucoma eye drops and concluded that prostaglandin analogs were most likely to induce hyperemia.

Preclinical trials indicated a high frequency of ripasudil-related hyperemia post-instillation^4,8,9^. In previous clinical trials, conjunctival hyperemia was apparently induced following ripasudil instillation. Tanihara *et al*. performed a phase 1 clinical trial and evaluated dose-dependent adverse events and efficiency for 50 healthy volunteers (placebo, 0.05%, 0.1%, 0.2%, 0.4%, and 0.8%) at 0.5, 2, 4, 8, and 9 h post-instillation^4^. Hyperemia in bulbar conjunctiva was induced at 30 min post-instillation and disappeared at 4 h post-instillation of 0.4% ripasudil ophthalmic solution in all 8 healthy volunteers. The safety and the effectiveness of ripasudil were confirmed in preclinical phase 2 and 3 clinical trials of patients with glaucoma^8,9^. They also reported that conjunctival hyperemia was the most frequent adverse event following instillation of ripasudil in participants with glaucoma, as well as healthy participants. Conjunctival hyperemia induced by 0.4% ripasudil ophthalmic solution occurred in 65.3% (phase 2) at 1 h post-instillation and in around 60% (phase 3 studies) at 2 h post-instillation^8,9^.

There have been two reports of conjunctival hyperemia post-instillation of 0.4% ripasudil ophthalmic solution. According to a report from Inoue’s group, conjunctival hyperemia developed in all 40 healthy participants 15 min post-instillation of 0.4% ripasudil ophthalmic solution, and conjunctival hyperemia disappeared in 92.5% of participants by 2 h post-instillation^10^. Terao *et al*. performed a more detailed analysis^5^ and demonstrated that conjunctival hyperemia peaked at approximately 5–15 min after ripasudil administration in all 50 healthy participants. Hyperemia induced by ripasudil was moderately severe, and the symptoms resolved within 2 h, thus elucidating the time-course of conjunctival hyperemia induced by ripasudil in healthy volunteers. However, similar information was not previously available for patients with glaucoma. Thus, we sought to evaluate the offset of conjunctival hyperemia induced by 0.4% ripasudil ophthalmic solution in patients with glaucoma who were already receiving other (non-ripasudil) anti-glaucoma eye drops.

Clinical grades for conjunctival hyperemia were evaluated in patients with glaucoma, post-instillation of 0.4% ripasudil ophthalmic solution. Our results indicated that conjunctival hyperemia was induced in 84%, and hyperemia disappeared within 2 h in 83.3%.

Of the 50 participants, there were 8 cases where clinical grade did not increase at 10 min post-instillation of ripasudil, compared with baseline. In 7 of these 8 cases, baseline clinical scores were 2 to 3, indicating moderate hyperemia. This high baseline level of conjunctival hyperemia may be the reason that we did not observe changes in clinical grades in these participants.

In some cases, conjunctival hyperemia did not disappear over 3 h. In this study, the frequency of conjunctival hyperemia induced by ripasudil was 84%, which was less than the previously reported data from healthy participants, which showed a 100% incidence^5^. Previous frequencies of hyperemia were 65.3% (phase 2) and around 60% (phase 3 studies)^8,9^. This discrepancy may be due to differences in our experimental protocols (i.e., in the preclinical trial, where evaluations were performed 1 to 2 h post-instillation). In this clinical study, the frequency of hyperemia remaining at 1 h post-instillation was 52.4%, which is close to the data of the previous clinical trial.

In the group with the offset time within 60 min, the proportion of patients who had been treated with PG Mono was significantly higher than that with β-blocker. In contrast, in the group with the offset time of 180 min or over, the proportion of patients who had been treated with β-blocker was significantly higher than that with PG Mono. These data may be interpreted that β-blocker instillation delays disappearance of hyperemia. However, the sample size was too small to draw such a conclusion, although the difference was statistically significant.

There were no significant trends for clinical grade increases and reduction of IOP. The correlation between the clinical grades and pixel coverage, generated by the hyperemia analysis software, showed a monotonic tendency to increase over time.

Regarding changes in hyperemia over time, in this trial baseline clinical grade values and pixel coverage values were higher than healthy subject data^5^. Terao *et al*. found an average baseline score of 0.19, in contrast to our result of 1.06 for clinical grade. Similarly, baseline pixel coverage value was 4.27 in Terao’s report^5^, compared to our value of 9.86. These data may be due to the fact that anti-glaucoma eye drops were previously used by our study participants. However, similar to Terao’s data^5^, the clinical grade and pixel coverage values were significantly decreased, compared to peak values.

In conclusion, conjunctival hyperemia, induced by 0.4% ripasudil instillation, appeared within 10 min post-instillation in patients with glaucoma who were previously treated with anti-glaucoma eye drops. Moreover, hyperemia resolved within 2 h post-instillation, indicating that the hyperemia, induced by ripasudil, was transient in this population.

## Methods

This was a multicenter, prospective, interventional, non-randomized open-label study received approval from the Institutional Review Board of Kochi Medical School, Hiroshima University, and Saneikai Tsukazaki Hospital (Clinical trial registration number: 27–75, date of registration: 16/09/2015). The study conformed to Declaration of Helsinki standards. Potential participants for the clinical trial were provided with comprehensive information regarding the study protocol (Supplementary Methods), and written informed consent was obtained before entry to the study. This clinical study was registered with UMIN (code: #000019565, date of registration: 30/10/2015).

### Procedures

Slit lamp photographs and IOP measures were performed between 12:00 and 14:00 before instillation as baseline and at 10, 60, 90, 120, 180 min after a single instillation of ripasudil. Binocular IOP values were compared and the eye with higher IOP value was selected at baseline. If the IOP value was the same in both eyes, the right eye was selected. The photographic method was held constant throughout this study, and the photographs were stored as JPEG images. In each participant, photographs of the bulbar conjunctiva on the temporal side were taken with a slit lamp (SL-D7; TOPCON, Tokyo, Japan). The angle between the slit lamp and the microscope arm was set at 30°. The camera flash light was adjusted to level one. The slit width was set 20 mm, and the objective magnification was set at 10. The diffuser of the slit lamp was used, and the resultant photographs were evaluated either by clinical grading or by automated hyperemia analysis software.

### Clinical grade of conjunctival hyperemia

**Figure 5:**
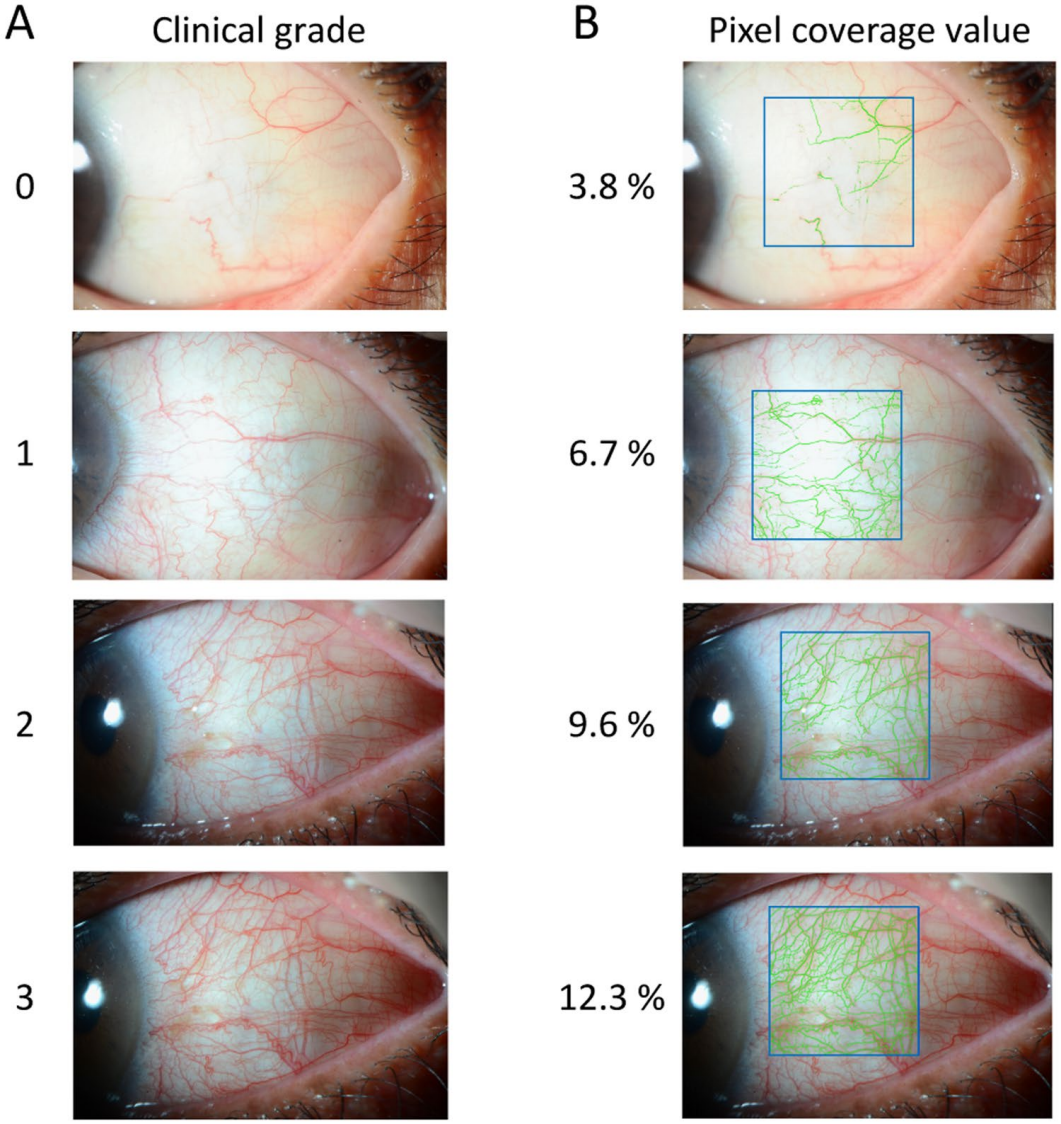
Clinical grade and pixel coverage value of conjunctival hyperemia. Typical photograph of each clinical grade is shown (**A**). The software calculates the proportion of the conjunctival blood vessels as the pixel coverage. The pixel coverage value was automatically calculated for the closed square area (vertical 60% and horizontal 40%). When processing the photographs shown in (**A**) to the analysis software, blood vessels are drawn in the green area (**B**).

Clinical grade of conjunctival hyperemia occurred at the temporal bulbar conjunctiva was evaluated in each case. The clinical grade scores were 0 (none: no hyperemia of the bulbar conjunctiva), 1 (mild: the dilation of a few conjunctival blood vessels), 2 (moderate: the dilation of some conjunctival blood vessels), and 3 (severe: the dilation of many conjunctival blood vessels), based on Japanese guidelines for allergic conjunctival disease^11^. Clinical grades were evaluated by 3 medical ophthalmic physicians, using the photographs taken at each of the 6 time points. A representative photograph is presented as Fig. 5A. We selected the most frequent grade value generated by the 3 physicians. When the scores differed among the 3 physicians, we selected the maximum value.

### Evaluating pixel coverage on conjunctival vessels using conjunctival hyperemia analysis software

The photographs were processed using the software program developed by our group^12,13^. Briefly, this software calculates the proportion of the blood vessels in the conjunctiva as the pixel percent coverage. The photographs were transferred to the software to calculate pixel coverage. To detect blood vessels, the green component of the RGB color model was used, and we adjusted the green value to accurately detect blood vessels within the region of interest (ROI). The pixel coverage was calculated by dividing the frequency of conjunctival blood vessel pixels by the total pixel frequency. For each selected region, the extent of hyperemia was determined by the percentage of pixelated blood vessels in the ROI. The ROI was depicted as the square, bordered by a blue line, with a vertical width of 60% and a horizontal width of 40%, using analysis software. Figure 5B depicts an automatically-created representative photograph. Blood vessels are displayed within the green area, and the blood vessel occupancy rate is shown in the ROI (Fig. 5B). The pixel coverage of conjunctival blood vessels was evaluated by 3 doctors using the ROIs within photographs taken at each of the 6 time points. We selected the most frequent pixel coverage value, as determined by 3 doctors. When the value differed among the 3 doctors, we selected the maximum value so as to not underestimate incidence of hyperemia.

### IOP measurements using the iCare TA01i tonometer

Our method for measuring IOP was selected in order to not affect conjunctival hyperemia. Although the “gold standard” evaluation method is the Goldmann applanation tonometer; however, use of anesthesia eye drops may affect hyperemia. Therefore, we selected a technique that did not require anesthesia and measured the IOP, in the sitting position, using an iCare TA01i tonometer^14^.

### Study population

We enrolled adult participants (older than 20y) with open-angle glaucoma or OHT, who needed additional eye drop treatment, and had never been treated with ripasudil ophthalmic solution. The inclusion and exclusion criteria for this study are outlined in Table 3.

**Table 3:** Inclusion and exclusion criteria.

**Inclusion criteria**
• The participants were (males or females) over 20 years old with open angle glaucoma or OHT, poorly controlled by ophthalmic solutions and requiring additional treatment
• Prior treatment with one or more of prostaglandin (PG) analogs, and/or beta-blockers, and/or acetazolamide
• No history of treatment with ripasudil ophthalmic solution
**Exclusion criteria**
• Participants with hypersensitivity against ripasudil hydrochloride hydrate, anhydrous dihydrogen phosphate sodium, glycerin, sodium hydroxide, or concentrated benzalconium chloride liquid
• Less than −12 dB of mean deviation (MD) value
• Women of childbearing potential who were pregnant, nursing, or planning a pregnancy
• Secondary glaucoma (excluding exfoliation glaucoma)
• Angle closure glaucoma
• Refract value: Less than −9.0 D, More than +9.0 D
• Participants whose intra ocular pressure cannot be measured by the iCare Tonometer
• Participants with traumatic injuries
• Participants with ocular inflammation of the anterior segment
• Participants who cannot tolerate photo slit
• Participants having with histories of surgeries during the past 6 months.
• Users of contact lenses
• Individuals deemed unsuitable for this trial by doctors

### Primary and secondary endpoints

The primary endpoint was median offset time for hyperemia, as determined by clinical grade scoring. The hyperemia offset time was determined as the time-point when clinical grade score increased by instillation of ripasudil returned to the baseline score. Our secondary endpoints were relationships among clinical grade changes over time, IOP, and pixel coverage on conjunctival blood vessels within 180 min post-instillation. The correlations between clinical grade and pixel coverage, and clinical grade and IOP reduction were statistically analyzed.

### Statistical analyses

Fifty cases were selected for the purpose of exploratory data analysis and determination of feasibility. The statistical analyses were performed using the statistical package R, version 3.4.1 (R Foundation for statistical Computing, Vienna, Austria). For the primary endpoint, median offset of conjunctival hyperemia, we used a (two-tailed) 95% confidence interval, as follows. The lower bound was the [(n + 1)/2 − √n]-th value counted from the minimum value. The upper bound was the [(n + 1)/2 − √n]-th value counted from the maximum value. (The value [(n + 1)/2 − √n] was the whole number part of (n + 1)/2 − √n.) Ultimately, 42 participants were selected for final analyses. Eight participants with no increases in clinical grade post-instillation, relative to baseline, were excluded from the primary endpoint. Secondary endpoints were assessed by using repeated-measure analysis of variance (ANOVA) for all 50 participants. Paired-*t* test was carried out when it was judged that there was a significant difference between the two groups using ANOVA. The data were expressed as means ± SDs. *P* values * < 0.05 and ** < 0.01 were considered statistically significant. The correlation between the clinical grade and the value of pixel coverage of the blood vessels, and clinical grade and IOP, were subjected to statistical analyses using the Jonckheere-Terpstra Trend Test.

## Supplementary information

The protocol
