# Supplementary material for: Evaluation of offset of conjunctival hyperemia induced by a Rho-kinase inhibitor; 0.4% Ripasudil ophthalmic solution clinical trial
Source: Sci Rep. 2019 Mar 6;9:3755. doi: 10.1038/s41598-019-40255-9 (PMC6403378; doi:10.1038/s41598-019-40255-9)
Supplement: Supplementary file 1 — The protocol [file 41598_2019_40255_MOESM1_ESM.doc]

# **Trial title**

Evaluation of offset of conjunctival hyperemia induced by a Rho-kinase inhibitor; 0.4% Ripasudil ophthalmic solution clinical trial

# **Investigators**

Chief investigator: Atsuki Fukushima; Professor

Department of Ophthalmology and Visual Science, Kochi Medical School, Kochi University, Kohasu, Oko-cho, Nankoku-city, Kochi 783-8505 Japan

Tel.: +81-888-80-2391

Fax: +81-888-80-2392

Emi Sakamoto

Department of Ophthalmology and Visual Science, Kochi Medical School, Kochi University, Nankoku City, Kochi, Japan

Waka Ishida

Department of Ophthalmology and Visual Science, Kochi Medical School, Kochi University, Nankoku City, Kochi, Japan

Tamaki Sumi

Department of Ophthalmology and Visual Science, Kochi Medical School, Kochi University, Nankoku City, Kochi, Japan

Tatsuma Kishimoto

Department of Ophthalmology and Visual Science, Kochi Medical School, Kochi University, Nankoku City, Kochi, Japan

Kentaro Tada

Department of Ophthalmology and Visual Science, Kochi Medical School, Kochi University, Nankoku City, Kochi, Japan

Ken Fukuda

Department of Ophthalmology and Visual Science, Kochi Medical School, Kochi University, Nankoku City, Kochi, Japan

Tsuyoshi Yoneda

Department of Ophthalmology, Kawasaki University of Medical Welfare, Kurashiki City, Okayama, Japan

Hajime Kuroiwa

Integrated Center for Advanced Medical Technologies, Kochi Medical School, Kochi University, Nankoku City, Kochi, Japan

Etsuko Terao

Department of Ophthalmology, Saneikai Tukazaki Hospital, Himeji City, Hyogo, Japan

Yasuko Fujisawa

Department of Ophthalmology, Saneikai Tukazaki Hospital, Himeji City, Hyogo, Japan

Shunsuke Nakakura

Department of Ophthalmology, Saneikai Tukazaki Hospital, Himeji City, Hyogo, Japan

Koji Jian

Department of Ophthalmology and Visual Sciences, Graduate School of Biomedical Sciences, Hiroshima University, Hiroshima City, Hiroshima, Japan

Hideaki Okumichi

Department of Ophthalmology and Visual Sciences, Graduate School of Biomedical Sciences, Hiroshima University, Hiroshima City, Hiroshima, Japan

Yoshiaki Kiuchi

Department of Ophthalmology and Visual Sciences, Graduate School of Biomedical Sciences, Hiroshima University, Hiroshima City, Hiroshima, Japan

# **General information**

**Title registration at UMIN**: Evaluation of conjunctival hyperemia induced by Glanatec ophthalmic solution 0.4%

**UMIN Number**：UMIN000019565, date of registration; 30/10/2015

**Funder**: Kowa Company, Ltd. 6-29, Nishiki 3-chome, Naka-ku, Nagoya-city, Aichi, Japan

# **Project summary**

The purpose of this study is to investigate the onset, offset, and kinetic changes of conjunctival hyperemia induced by 0.4% ripasudil ophthalmic solution; Glanatec ophthalmic solution in participants with open-angle glaucoma or ocular hypertension (OHT). Ripasudil-naïve adults (n=50) with glaucoma who were undergoing eye drop treatment are enrolled. Slit lamp photographs and IOP measures are performed in one eye at baseline and 10, 60, 90, 120, 180 min after instillation of ripasudil. The resultant photographic images are objectively evaluated by 3 ophthalmic physicians using conjunctival hyperemia-analyzing software. Dependent variables are conjunctival hyperemia offset, clinical changes, IOP, and pixel coverage of conjunctival vessels post-instillation. We determine correlations between clinical grade and pixel coverage, and clinical grade and IOP reduction. In this clinical study, it is possible to clarify the onset, offset and kinetic changes of conjunctival hyperemia, induced by 0.4% ripasudil ophthalmic solution, in patients with open-angle glaucoma or OHT.

# **Rationale & background information**

Glaucoma can cause irreversible blindness, so numerous anti-glaucoma eye drops have been developed. Unfortunately, many patients with glaucoma still suffer from progressive visual defects and vision disorders. The most important risk factor for progression is intraocular pressure (IOP). Rho-associated protein kinase (ROCK) inhibitors lower IOP and are also associated with adverse events; most frequently, conjunctival hyperemia. In December 2014, ripasudil hydrochloride hydrate, a selective ROCK inhibitor (GLANATEC ophthalmic solution 0.4%) was launched as an anti-glaucoma treatment in Japan. A recent clinical trial with healthy participants investigated changes in conjunctival hyperemia and reduction of IOP within 2 h post-instillation of 0.4% ripasudil. Here, conjunctival hyperemia peaked 5-15 min post-instillation and resolved within 2 h. IOP values decreased between 30 min and 2 h post-instillation, regarding healthy participants. However, ripasudil-induced changes in conjunctival hyperemia over time have not been investigated in patients with glaucoma, who were previously treated with anti-glaucoma eye drops, other than ripasudil.

# **References (of literature cited in preceding sections)**

1. Honjo, M., et al. Effects of Rho-associated protein kinase inhibitor Y-27632 on intraocular pressure and outflow facility. *Invest. Ophthalmol. Vis. Sci.* **42**, 137-144 (2001).
2. Kameda, T., et al. The effect of Rho-associated protein kinase inhibitor on monkey Schlemm’s canal endothelial cells. *Invest. Ophthalmol. Vis. Sci.* **53**, 3092-3103 (2012).
3. Inoue, T., Tanihara, H. Rho-associated kinase inhibitors: a novel glaucoma therapy. *Prog. Rein. Eye Res.* **37**, 1-12 (2013).
4. Tanihara, H., et al. K-115 Clinical Study Group. Phase 1 clinical trials of a selective Rho-kinase inhibitor, K-115. *JAMA Ophthalmol.* **131**, 1288-1295 (2013). Erratum in: *JAMA Ophthalmol.* **132**, 787 (2014).
5. Terao, E., et al. Time course of conjunctival hyperemia induced by a Rho-kinase inhibitor anti-glaucoma eye drop: Ripasudil 0.4%. *Curr. Eye Res.* **42**, 738-742 (2017)*.*
6. Japanese Ocular Allergology Society [Guidelines for the clinical management of allergic conjunctival disease (2nd edition)]. *Jpn. Ophthalmol.***114**, 831-870 (2010).
7. Yoneda, T., et al. Automated hyperemia analysis software: reliability and reproducibility in healthy subjects. *Jpn. J. Ophthalmol.***56**, 1-7 (2012).
8. Sumi, T., et al. Development of automated conjunctival hyperemia analysis software. *Cornea.* **32**, 52-5 (2013).
9. Pakrou, N., Gray, T., Mills, R., Landers, J., Craig, J. Clinical comparison of the Icare tonometer and Goldmann applanation tonometry: *J. Glaucoma.* **17**, 43-47 (2008).
10. Honrubia, F., García-Sánchez, J., Polo, V., de la Casa, JM., Soto, J. Conjunctival hyperaemia with the use of latanoprost versus other prostaglandin analogues in patients with ocular hypertension or glaucoma: a meta-analysis of randomised clinical trials. *Br. J. Ophthalmol.* **93**, 316-321 (2009).
11. Yanagi, M., et al. Association between glaucoma eye drops and hyperemia. *Jpn. J. Ophthalmol.* **60**, 72-77 (2016).
12. Tanihara H, et al. K-115 Clinical Study Group. Phase2 randomaized clinical study of a Rho kinase inhibitor, K-115, in primary open-angle glaucoma and ocular hypertension. *Am. J. Ophthalmol.* **156**, 731-736 (2013).
13. Tanihara, H., et al. K-115 Clinical study group additive intraocular pressure-lowering effects of the Rho Kinase Inhibitor Ripasudil (K-115) combined with Timolol or Latanoprost: A report of 2 randomized clinical trials. *JAMA Ophthalmol.* **133**, 755-761 (2015).
14. Inoue, K., et al. Conjunctival hyperemia post-instillation of one drop of ripasudil ophthalmic solution in volunteers: *Jpn. J. Ophthalmol.***71**, 103-108 (2017).

# **Study goals and objectives**

The primary endpoint of this study is to evaluate median offset time for conjunctival hyperemia induced by 0.4% ripasudil ophthalmic solution in patients with glaucoma. The evaluation is based on clinical grade scoring. The hyperemia offset time was determined as the time-point when clinical grade score increased by instillation of ripasudil returned to the baseline score. Additionally, the relationships among clinical grade changes over time, IOP, and pixel coverage on conjunctival blood vessels within 180 min post-instillation will also be evaluated for the secondary endpoints. The correlations between clinical grade and pixel coverage, and clinical grade and IOP reduction will be statistically analyzed. Furthermore, by using conjunctival hyperemia analyzing software, evaluation of conjunctival hyperemia will be more objective.

# **Study Design**

**Study design:** Multicenter, prospective, interventional, non-randomized open-label study.

**Trial characteristic:** Exploratory

**Purpose of intervention:** Treatment

**Type of intervention: Medicine:** At the beginning of trial, one drop of 0.4% ripasudil solution will be instilled to the subjective eye.

**Target sample size:** 50

This clinical trial was not based on a statistical hypothesis test, and 50 cases were taken as the target number of cases from the feasibility of this trial.

**Inclusion criteria:**

1. The participants were (males or females) over 20 years old with open angle glaucoma or OHT, poorly controlled by ophthalmic solutions and requiring additional treatment
2. Prior treatment with one or more of prostaglandin (PG) analogs, and/or beta-blockers, and /or acetazolamide
3. No history of treatment with ripasudil ophthalmic solution

**Exclusion criteria:**

1. Participants with hypersensitivity against ripasudil hydrochloride hydrate, anhydrous dihydrogen phosphate sodium, glycerin, sodium hydroxide, or concentrated benzalconium chloride liquid
2. Less than -12dB of mean deviation (MD) value
3. Women of childbearing potential who were pregnant, nursing, or planning a pregnancy
4. Secondary glaucoma (excluding exfoliation glaucoma)
5. Angle closure glaucoma
6. Refract value: Less than -9.0D, More than +9.0D
7. Participants whose intra ocular pressure cannot be measured by the iCare Tonometer
8. Participants with traumatic injuries
9. Participants with ocular inflammation of the anterior segment
10. Participants who cannot tolerate photo slit
11. Participants having with histories of surgeries during the past 6 months
12. Users of contact lenses
13. Individuals deemed unsuitable for this trial by doctors

# **Methodology Procedures**

Slit lamp photographs and IOP measures were performed between 12:00 and 14:00 before instillation as baseline and at 10, 60, 90, 120, 180 min after a single instillation of ripasudil. Binocular IOP values were compared and the eye with higher IOP value was selected at baseline. If the IOP value was the same in both eyes, the right eye was selected. The photographic method was held constant throughout this study, and the photographs were stored as JPEG images. In each participant, photographs of the bulbar conjunctiva on the temporal side were taken with a slit lamp (SL-D7; TOPCON, Tokyo, Japan). The angle between the slit lamp and the microscope arm was set at 30°. The camera flash light was adjusted to level one. The slit width was set 20 mm, and the objective magnification was set at 10. The diffuser of the slit lamp was used, and the resultant photographs were evaluated either by clinical grading or by automated hyperemia analysis software.

**Clinical grade of conjunctival hyperemia**

Clinical grade of conjunctival hyperemia occurred at the temporal bulbar conjunctiva was evaluated in each case. The clinical grade scores were 0 (none: no hyperemia of the bulbar conjunctiva), 1 (mild: the dilation of a few conjunctival blood vessels), 2 (moderate: the dilation of some conjunctival blood vessels), and 3 (severe: the dilation of many conjunctival blood vessels), based on Japanese guidelines for allergic conjunctival disease.^6^ Clinical grades were evaluated by 3 medical ophthalmic physicians, using the photographs taken at each of the 6 time points. We selected the most frequent grade value generated by the 3 physicians. When the scores differed among the 3 physicians, we selected the maximum value.

**Evaluating pixel coverage on conjunctival vessels using conjunctival hyperemia analysis software**

The photographs were processed using the software program developed by our group. Briefly, this software calculates the proportion of the blood vessels in the conjunctiva as the pixel percent coverage. The photographs were transferred to the software to calculate pixel coverage. To detect blood vessels, the green component of the RGB color model was used, and we adjusted the green value to accurately detect blood vessels within the region of interest (ROI). The pixel coverage was calculated by dividing the frequency of conjunctival blood vessel pixels by the total pixel frequency. For each selected region, the extent of hyperemia was determined by the percentage of pixelated blood vessels in the ROI. The ROI was depicted as the square, bordered by a blue line, with a vertical width of 60% and a horizontal width of 40%, using analysis software. The pixel coverage of conjunctival blood vessels was evaluated by 3 doctors using the ROIs within photographs taken at each of the 6 time points. We selected the most frequent pixel coverage value, as determined by 3 doctors. When the value differed among the 3 doctors, we selected the maximum value so as to not underestimate incidence of hyperemia.

**IOP measurements using the iCare TA01i tonometer**

Our method for measuring IOP was selected in order to not affect conjunctival hyperemia. Although the “gold standard” evaluation method is the Goldmann applanation tonometer; however, use of anesthesia eye drops may affect hyperemia. Therefore, we selected a technique that did not require anesthesia and measured the IOP, in the sitting position, using an iCare TA01i tonometer.

**Statistical analyses**

The statistical analyses were performed using the statistical package R, version3.4.1 (R Foundation for statistical Computing, Vienna, Austria). For the primary endpoint, median offset of conjunctival hyperemia, we used a (two-tailed) 95% confidence interval, as follows. The lower bound was the [(n+1)/2−
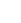
]-th value counted from the minimum value. The upper bound was the [(n+1)/2−
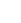
]-th value counted from the maximum value. (The value [(n+1)/2−
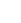
] was the whole number part of (n+1)/2−
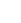
.) Participants with no increases in clinical grade post-instillation, relative to baseline, are excluded from the primary endpoint. Secondary endpoints were assessed by using repeated-measure analysis of variance (ANOVA) for all 50 participants. Paired-*t* test was carried out when it was judged that there was a significant difference between the two groups using ANOVA. The data were expressed as means ± SDs. *P* values *****< 0.05 and ****<** 0.01 were considered statistically significant. The correlation between the clinical grade and the value of pixel coverage of the blood vessels, and clinical grade and IOP, were subjected to statistical analyses using the Jonckheere-Terpstra Trend Test.

# **Safety Considerations**

The study conformed to Declaration of Helsinki standards. Potential participants for the clinical trial were provided with comprehensive information regarding the study protocol. This study was a trial with marketed product; phase 4 and an investigator- initiated clinical trial.

# **Follow-Up**

None

# **Data Management and Statistical Analysis**

In this clinical study, the Integrated Center for Advanced Medical Technologies of Kochi Medical School conducted data management, data reports, monitoring and statistical analysis. The data of research subject and electric case report were collected using Electric Data Capture (EDC) system.

**Monitoring matter:**

1. Number of registered cases
2. Eligibility of registered cases
3. Report of serious adverse events
4. Significant deviation from protocol
5. Progress of testing, safety, reliability
6. Compliance with ethical guidelines

# **Quality Assurance**

In order to ensure the reliability of this trial, an audit is carried out by auditors.

# **Expected Outcomes of the Study**

In this clinical trial, understanding the onset and offset of conjunctival hyperemia by 0.4% ripasudil ophthalmic solution makes it possible to know details of side effects.

# **Dissemination of Results and Publication Policy**

This clinical study was registered with UMIN (code #000019565, date of registration; 30/10/2015). After the clinical trial, we will present at academic conference and submit a paper. Upon publication, all of data should be adequately managed so that the subject is not specified.

# **Duration of the Project**

From 16/09/2015 to 30/06/2017

# **Problems Anticipated**

Conjunctival hyperemia, conjunctivitis, blepharitis and photophobia by taking photography

# **Project Management**

**The lead organization (coordinating center):** Kochi Medical School

**The principal investigator**

Atsuki Fukushima; Department of Ophthalmology, Kochi Medical School

The principal investigator should also be supportive and committed, and be available to take the lead on clinical or scientific issues.

**Responsible investigator:** Atsuki Fukushima

**Local treating physician:** Emi Sakamoto; Department of Ophthalmology, Kochi Medical School

**Clinical grades were evaluated by 3 medical ophthalmic physicians:**

Ken Fukuda; Department of Ophthalmology, Kochi Medical School

Kentaro Tada; Department of Ophthalmology, Kochi Medical School

Tatsuma Kishimoto; Department of Ophthalmology, Kochi Medical School

**The pixel coverage of conjunctival blood vessels was evaluated by 3 doctors:**

Waka Ishida; Department of Ophthalmology, Kochi Medical School

Tamaki Sumi; Department of Ophthalmology, Kochi Medical School

Tsuyoshi Yoneda; Department of Ophthalmology, Kawasaki University of Medical Welfare

**Data center, Monitoring, Statistical analyses:**

Hajime Kuroiwa; Integrated Center for Advanced Medical Technologies, Kochi Medical School

**The participating institutions:**

Department of Ophthalmology, Hiroshima University

**Responsible investigator:** Yoshiaki Kiuchi, Professor

**Local treating physician:** Hideaki Okumich, Koji Jian

Department of Ophthalmology, Saneikai Tukazaki Hospital

**Responsible investigator:** Shunsuke Nakakura

**Local treating physician:** Etsuko Terao, Yasuko Fujisawa

# **Ethics**

This study received approval from the Institutional Review Board of Kochi Medical School, Hiroshima University, and Saneikai Tukazaki Hospital. The study conformed to Declaration of Helsinki standards. Potential participants for the clinical trial were provided with comprehensive information regarding the study protocol, and written informed consent was obtained before entry to the study.

# **Informed Consent Forms**

The study conformed to Declaration of Helsinki standards. Potential participants for the clinical trial were provided with comprehensive information regarding the study protocol, and written informed consent was obtained before entry to the study.

# **Budget**

None

# **Other support for the Project**

The cost for this study will be provided by contract to Kochi Medical School with Kowa Company, Ltd.

# **Collaboration with other scientists or research institutions:** None

# **Links to other projects:** None

# **Curriculum Vitae of investigators:** None

# **Other research activities of the investigators:** None
